# Organization and Dynamics of the Red Blood Cell Band 3 Anion Exchanger SLC4A1: Insights From Molecular Dynamics Simulations

**DOI:** 10.3389/fphys.2022.817945

**Published:** 2022-02-25

**Authors:** Antreas C. Kalli, Reinhart A. F. Reithmeier

**Affiliations:** ^1^Leeds Institute of Cardiovascular and Metabolic Medicine and Astbury Center for Structural Molecular Biology, University of Leeds, Leeds, United Kingdom; ^2^Department of Biochemistry, University of Toronto, Toronto, ON, Canada

**Keywords:** Band 3, anion transport, red blood cell, membrane transporters, molecular dynamics simulations

## Abstract

Molecular dynamics (MD) simulations have provided new insights into the organization and dynamics of the red blood cell Band 3 anion exchanger (AE1, SLC4A1). Band 3, like many solute carriers, works by an alternating access mode of transport where the protein rapidly (10^4^/s) changes its conformation between outward and inward-facing states *via* a transient occluded anion-bound intermediate. While structural studies of membrane proteins usually reveal valuable structural information, these studies provide a static view often in the presence of detergents. Membrane transporters are embedded in a lipid bilayer and associated lipids play a role in their folding and function. In this review, we highlight MD simulations of Band 3 in realistic lipid bilayers that revealed specific lipid and protein interactions and were used to re-create a model of the Wright (Wr) blood group antigen complex of Band 3 and Glycophorin A. Current MD studies of Band 3 and related transporters are focused on describing the trajectory of substrate binding and translocation in real time. A structure of the intact Band 3 protein has yet to be achieved experimentally, but cryo-electron microscopy in combination with MD simulations holds promise to capture the conformational changes associated with anion transport in exquisite molecular detail.

## Introduction

Band 3, the red blood cell Cl^−^/HCO_3_^−^ anion exchanger 1 (AE1), is the founding member of the SLC4 family of bicarbonate transporters and plays an essential role in respiration (Alper, 2009; Cordat and Reithmeier, 2014; Jennings, 2021). In the tissues, carbon dioxide enters the red blood cell primarily by diffusion where it is hydrated by carbonic anhydrase II to from bicarbonate and a proton that is buffered by hemoglobin. The bicarbonate is rapidly (10^4^ ions/s/Band 3) transported out of the red blood cell by the 1.2 million copies of Band 3 in exchange for chloride, increasing the blood’s capacity to transport carbon dioxide as plasma bicarbonate. In the lung, bicarbonate enters the red blood cell by Band 3 in exchange for chloride where it is dehydrated by carbonic anhydrase II to form carbon dioxide and water; the carbon dioxide diffusing out of the red blood cells to be expired.

**Figure 1:**
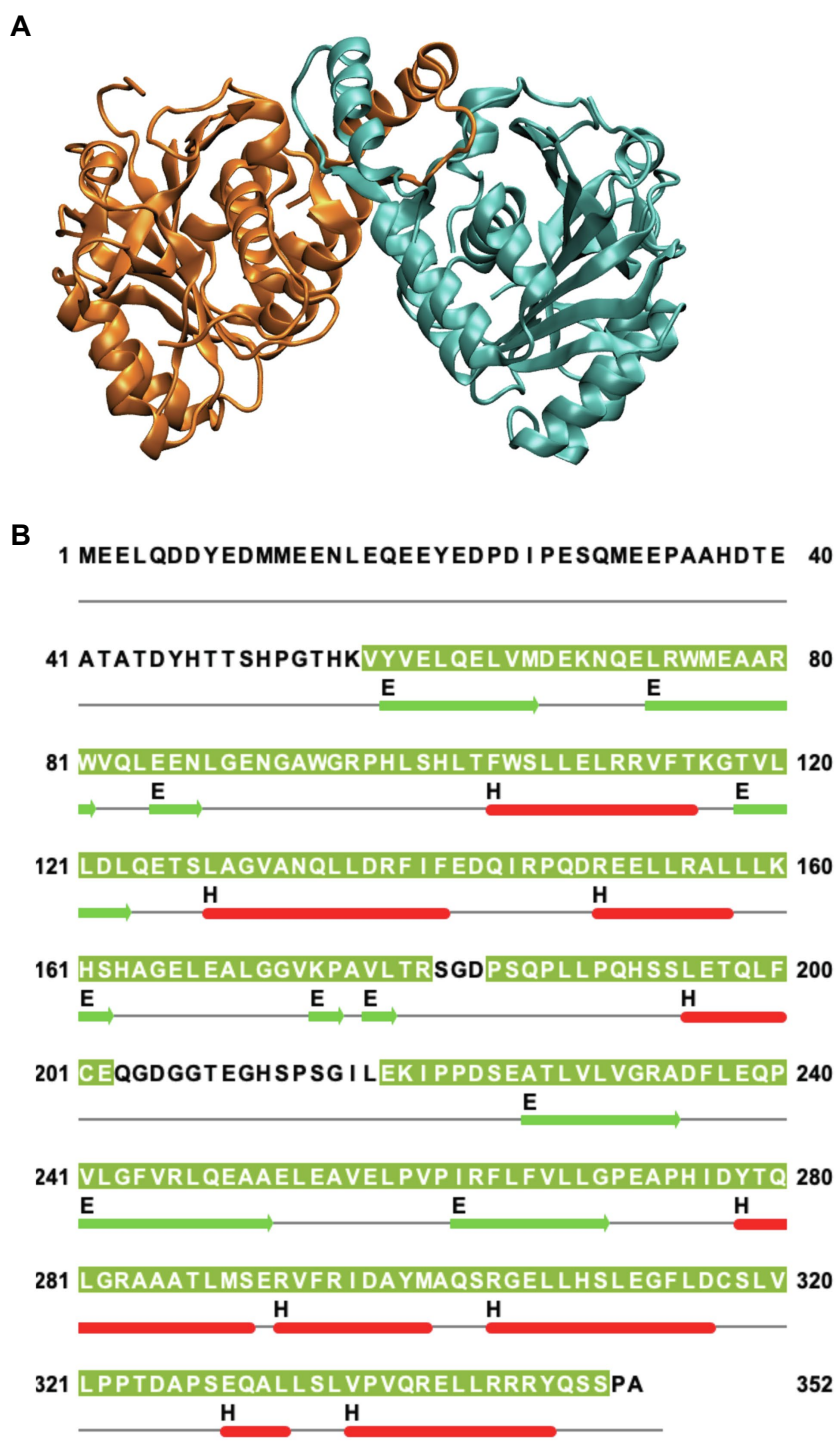
**(A)** Side view of the structure of the dimeric cytoplasmic domain of human Band 3 (PDB: 4KY9). The two subunits are shown in orange and cyan. **(B)** Corresponding sequence of human Band 3 cytoplasmic domain. The regions for which structural data exist for the Band 3 cytoplasmic (based on chain A of the 4KY9 structure in the Protein Data Bank) are indicated by olive green background. Helical (H) and β-sheet (E) regions are shown in red and green, respectively. Cysteine residues are located at C201 and C317. N-terminal disordered region (Residues 1–54) is not present in this structure. This figure was created using Jalview.

Human Band 3 is composed of 911 amino acids (Lux et al., 1989) organized into two major structural and functional domains (Steck et al., 1976). The amino-terminal cytoplasmic domain (Residues 1–360) can be released from the membrane by mild trypsin cleavage at Lys360 (Steck et al., 1971). This fragment can be readily expressed in *Escherichia coli* facilitating structural studies (Wang et al., 1992). Early work from the Low lab revealed a compact dimeric structure of this domain under low pH conditions (Zhang et al., 2000). A similar structure (Figure 1A) of a truncated version of the cytoplasmic domain missing the amino-terminal disordered acidic region was determined at neutral pH (Shnitsar et al., 2013). The isolated cytoplasmic domain of Band 3 is dimeric and undergoes dramatic pH-dependent conformational changes forming a more compact structure at low pH as revealed by the crystal structure (Zhou and Low, 2001).

Band 3 contains cysteine residues at residues 201 and 317 in the cytoplasmic domain (Figure 1B) that can be crosslinked to each other at neutral pH forming a covalent intermolecular dimer (Steck, 1972; Reithmeier and Rao, 1979). Interestingly, these two residues can be crosslinked intramolecularly to each other within each monomer in the isolated cytoplasmic domain. In the crystal structure of the isolated cytoplasmic domain, Cys 201 and Cys317 are ~29 Å apart in each monomer and ~20 Å apart across the dimer. This indicates that there is considerable conformational dynamics of the cytoplasmic domain. Perhaps, MD simulations can reveal the nature and scope of these conformational dynamics in the context of the isolated domain and the intact protein.

The cytoplasmic domain provides the site of interaction of Band 3 with hemoglobin, glycolytic enzymes, and the cytoskeleton (Low, 1986). Protein 4.2 stabilizes the interaction of Band 3 with ankyrin. The ankyrin binding site has been localized to a hairpin loop at residues 175–185 with contributions from residues 63–73 (Stefanovic et al., 2007). It may be possible to form a complex of the interacting portion of ankyrin with Band 3 for structural studies. Two Band 3 supramolecular complexes exist in red blood cells: the Band 3-ankyrin complex and the junctional complex (Bruce et al., 2003). The ankyrin complex not only includes Band 3, Glycophorin A, and protein 4.2 but also an association with the Rh complex (Rh-associated glycoprotein, Rh polypeptides, CD47, and LW), while the junctional complex includes Band 3, Glycophorin C, p55, protein 4.1, adducin, dematin, and GLUT1; both complexes provide linkages to the spectrin-actin cytoskeleton (Mankelow et al., 2012). Cryo-electron microscopy may be ideally suited to examine the structure of such supramolecular complexes *in situ*.

**Figure 2:**
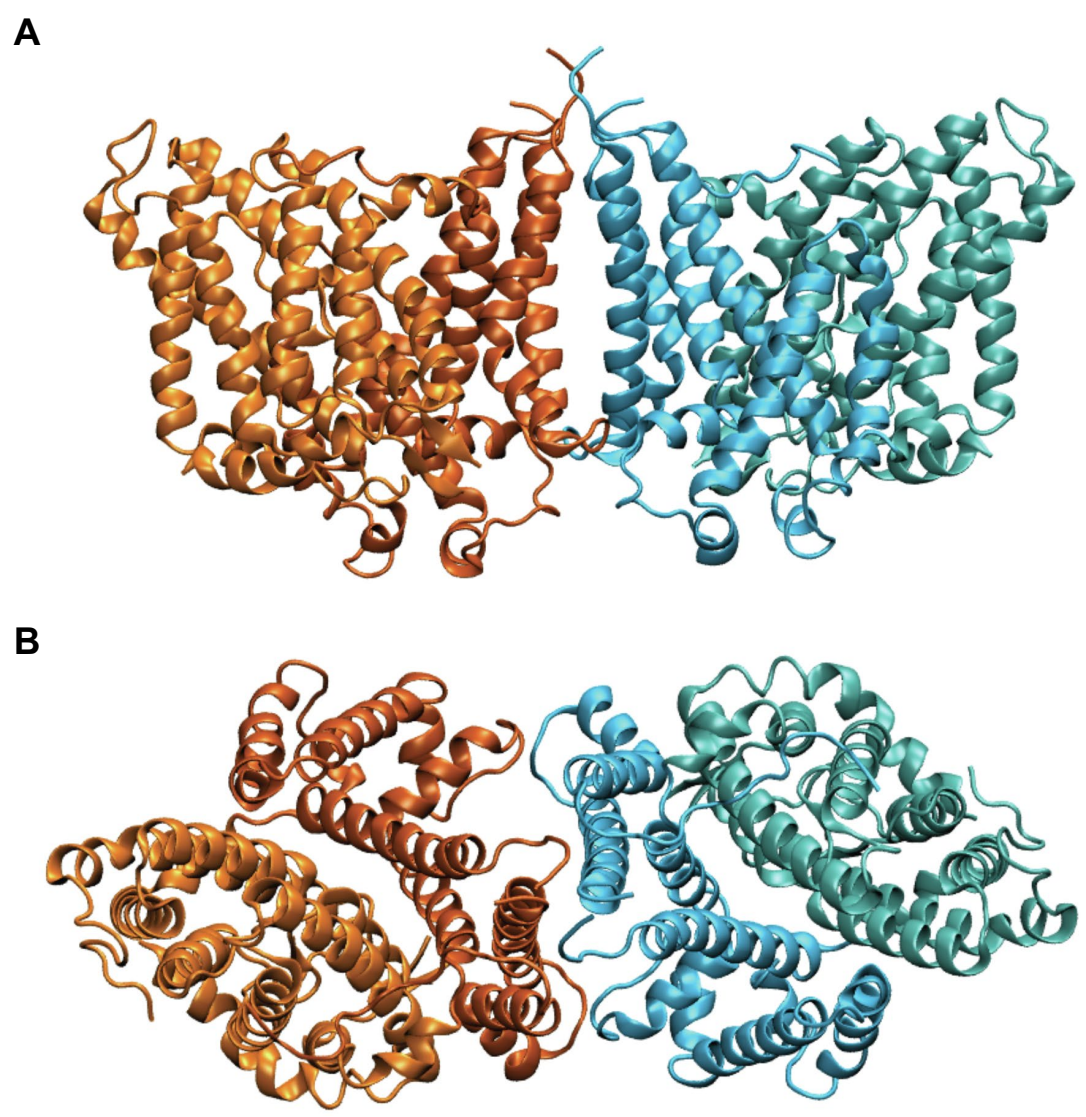
Side **(A)** and cytoplasmic **(B)** view of the structure of the dimeric membrane domain of human Band 3 (PDB: 4YZF). The two subunits are shown in orange and cyan. Two shades of orange and cyan show the gate and core domains of each subunit with the gate domains in darker shades at the dimer interface.

**Figure 3:**
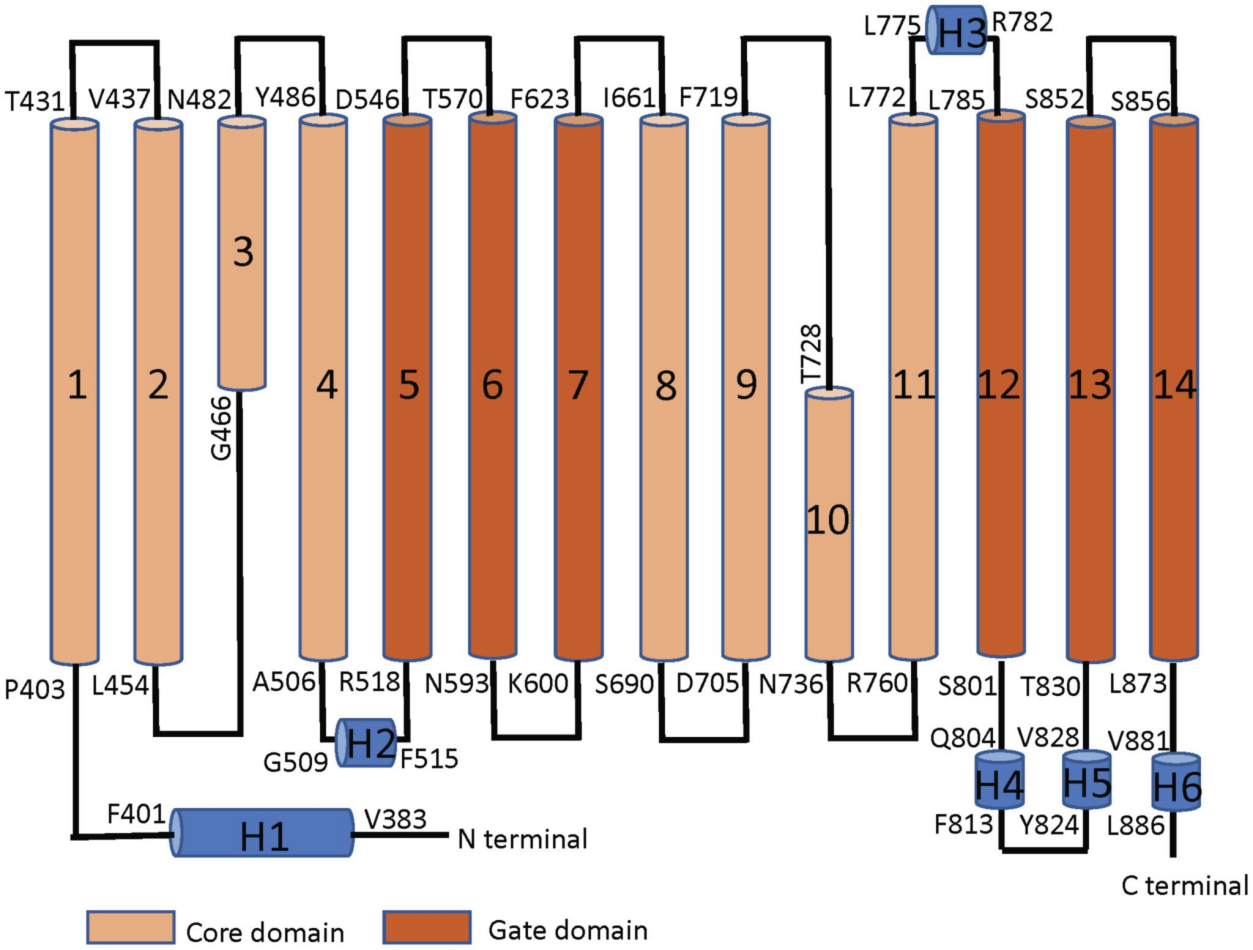
Linear topology of the membrane domain of human Band 3 showing its 14 TM structure with both the N− and C-termini on the cytoplasmic side of the membrane. The residues at the start and the end of the TM helices are shown. The TM segments in the core domain are shown in light brown and the segments in the gate domain in dark brown. TM3 and 10 are short helices that bind the substrate anion at their amino-terminal ends in the middle of the protein. Short helical regions joining TM segments are shown in blue (Adapted from Reithmeier et al., 2016).

Band 3 is a mixture of dimers and tetramers in the membrane and when solubilized in mild detergent solutions (Casey and Reithmeier, 1991). In contrast, the isolated membrane domain (residues 361–911) is exclusively a dimer (Reithmeier, 1979). The crystal structure of the dimeric membrane domain in the outward-facing conformation (Arakawa et al., 2015; Reithmeier et al., 2016; Figure 2) reveals a complex folding pattern with 14 transmembrane (TM) segments in a 7 + 7 TM inverted repeat common to members of the SLC4 family, the related SLC26 anion transporter family, and the unrelated SLC23 family of nucleobase transporters (Chang and Geertsma, 2017). Each subunit consists of two sub-domains, the gate domain (TM 5, 6, 7, 12, 13, and 14) at the dimer interface and the core domain (TM1, 2, 3, 4, 8, 9, 10, and 11) that contains the substrate binding site (Figure 3). The dimer is held together by interactions involving the extracellular ends of TM5 and 6. The Tanner lab (Groves et al., 1998) showed that complementary TM fragments of Band 3 (e.g. TM 1–2 + TM 3–14) representing the entire sequence could reassemble into functional units when expressed in Xenopus oocytes, including the unexpected finding that non-complementary TM1–5 and TM8–14 entirely missing TM6 and 7 was functional. The anion binding site is centrally located between the amino-termini of the short TM3 and TM10 segments with a positive charge contributed by Arg730 located in TM10. The short carboxyl-terminal tail of Band 3 interacts with carbonic anhydrase II forming the basis for a bicarbonate transport metabolon (Sterling et al., 2001).

## Band 3 Kinetics

Extensive kinetic studies have been carried out on anion transport in red blood cells (Knauf, 1979). Firstly, the affinity for anions is in the mM range, allowing a very rapid off-rate. Secondly, there is a single central anion binding site. Thirdly, Band 3 works by an alternating access mode of transport. Fourthly, the anion binding site is accessed by water-filled cavities. Fifthly, the empty carrier cannot undergo the transition enabling tight 1:1 exchange. Sixthly, anion binding reduces the energy barrier for the transition. Finally, anion exchange is very rapid (~10^4^ ions/s). The molecular details that allow for such rapid transport remain to be fully described.

Kinetic studies have also shown that SLC4A2 (AE2) operates by a simultaneous Cl^−^/Cl^−^exchange mechanism whereby the anion binding sites of both subunits must be occupied before translocation can occur (Restrepo et al., 1989). In contrast, Band 3 subunits work independently (Macara and Cantley, 1981) by a ping-pong mechanism (Falke and Chan, 1985). This suggests that the nature of the dimer interface may play a role in the mode of transport and its allosteric regulation in the SLC4 family of transporters.

The structural and functional studies of Band 3 described above have provided new insights into the mechanism of action of this transport protein. Yet, a complete molecular description of the conformational change associated with transport remains elusive. Furthermore, there are a number of inherited conditions, such as hereditary spherocytosis (HS), Southeast-Asian ovalocytosis (SAO), and distal renal tubular acidosis (dRTA) caused by mutations that affect Band 3 folding, trafficking, and function. A full understanding of the effect of these mutations on the structure and dynamics of Band 3 awaits further exploration. MD simulations are ideally suited to explore these issues since this powerful computational method provides a direct link between the structure and the conformational landscape accessible to the protein molecule. These dynamic insights will reveal how Band 3 works at the molecular level and how mutations associated with disease affect their ability to fold and function properly.

## Molecular DYNAMICS Simulations

Molecular dynamics (MD) simulations of membrane proteins in complex lipid bilayers, both at the coarse-grained and atomistic levels, have provided new insights into the organization and dynamics of these proteins (Marrink et al., 2019). Detergents are commonly used to solubilize, purify, and crystallize membrane proteins. Native membrane proteins however function within complex lipid bilayers. Some crystal structures of membrane proteins contain tightly-bound lipids, including cholesterol (Hunte and Richers, 2008). In the case of Band 3, removing these lipids results in destabilization of the protein and aggregation (Maneri and Low, 1989; Vince et al., 1997). Lipids play a key role in oligomerization of membrane transporters (Gupta et al., 2017).

Membrane transport proteins, like Band 3, are commonly found as oligomers, mostly as dimers. The oligomeric state allows for allosteric interactions between the subunits. In addition, the proper assembly of oligomers is a requirement for exit from the ER and may be part of protein quality control. As we will discuss, the dimeric structure of membrane transport proteins can provide a rigid scaffold to allow the relative movement of mobile elements associated with transport. In the case of Band 3 and transport proteins with a similar 7 + 7 inverted repeat structure, it is the gate domain that forms a rigid dimer interface and the core domain that binds substrate and undergoes movement to provide alternating access to the substrate binding site.

## The TM1 Signal Anchor

The first TM segment of Band 3 (Figure 3) acts as a signal anchor to target nascent Band 3 to the endoplasmic reticulum (ER) membrane (Popov et al., 1997). The first TM segment translocates the following TM2/3 region into the ER lumen with TM4 acting as a stop-transfer sequence (Cheung and Reithmeier, 2005). TM1 moves laterally from the translocon into the lipid bilayer, likely along with TM4. TM2 and 3 subsequently fold into the nascent structure. TM1-4 makes up part of the core domain. MD simulations showed that isolated TM1 can assume a native helical conformation similar to that found in the native protein (Fowler et al., 2017).

A nine-amino acid deletion (Ala400–Ala408) at the cytoplasmic interface region found in SAO Band 3 prevents the shortened TM segment from interacting properly with the lipid bilayer and forming a stable TM segment (Cheung et al., 2005). This feature accounts for the negative effect of the deletion on the folding and trafficking of SAO Band 3 (Moriyama et al., 1992; Sarabia et al., 1993). The SAO deletion removes Pro403 within a bend that connects the short H1 helix to the TM1 helix (Figure 3), which may result in a change in the relative orientation of the cytoplasmic and membrane domains. The more distal Pro419 places a flexible hinge in the middle of TM1 due to loss of backbone hydrogen bonds to residues 3 and 4 proximal, leaving the carbonyl groups at Ala415 and Ala416 free for other hydrogen-bonding interactions. Early NMR studies (Chambers et al., 1999) of synthetic peptides corresponding to TM1 revealed the role of proline residues in disrupting its helical structure and the effect of the SAO deletion. Despite these studies, the structural effect of the SAO mutation on the membrane domain of Band 3 has not been fully described. Current studies using MD simulations to construct a molecular model of SAO Band 3 will allow us to examine these details at the atomistic level.

## Band 3-Glycophorin A Interactions

**Figure 4:**
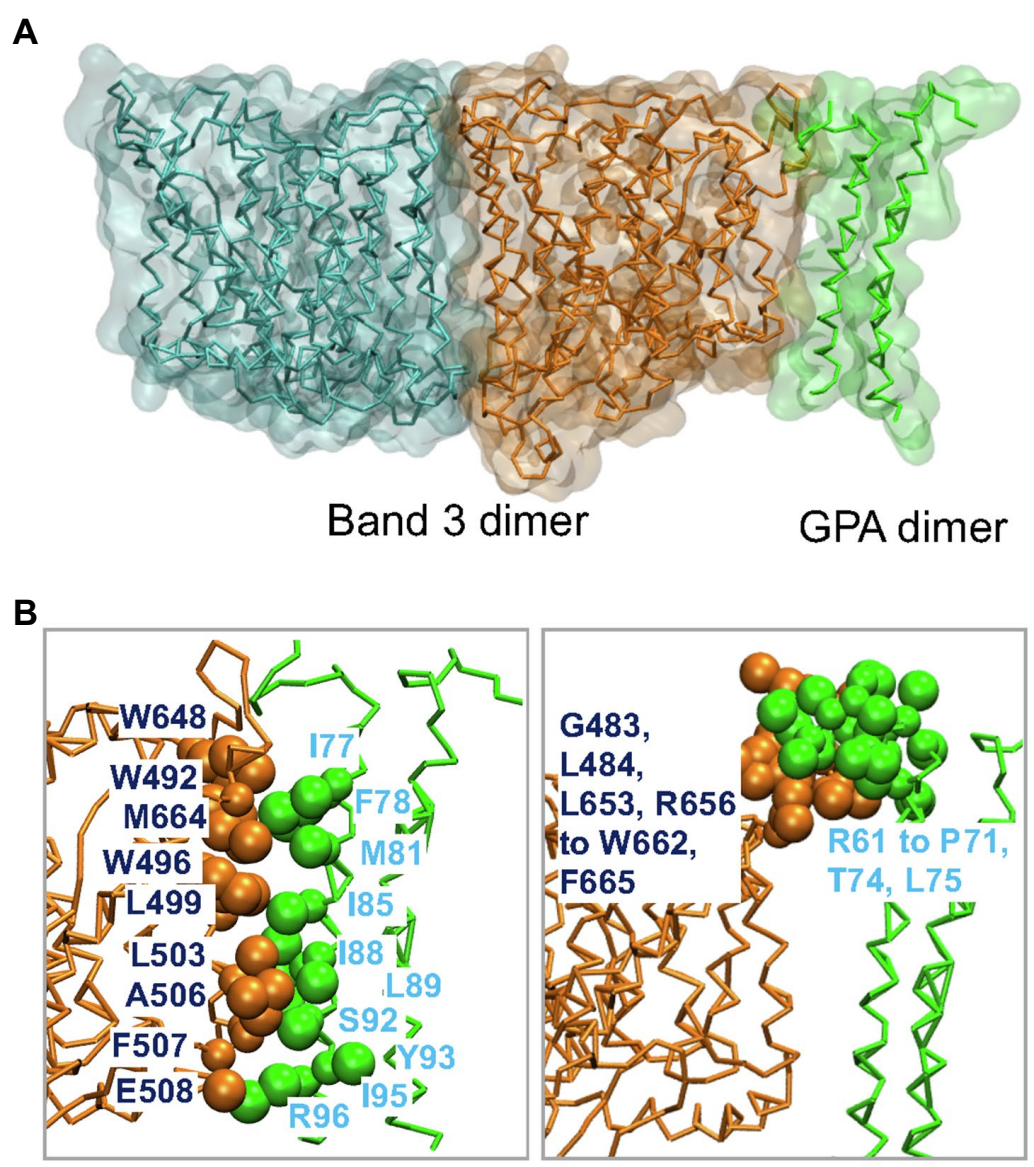
**(A)** Molecular dynamics (MD) simulation model of the interaction of the dimeric membrane domain of Band 3 shown in orange and cyan and the TM portion of the Glycophorin A (GPA) dimer shown in green. This snapshot is taken from one of the simulations in Kalli and Reithmeier (2018). **(B)** Contacts between Band 3 (orange) and Glycophorin A (green). Main residues in the interface between the TM segments of Band 3 and Glycophorin A are shown in VdW representation. See Kalli and Reithmeier (2018) for more details.

Band 3 interacts with Glycophorin A forming the Wright (Wr) blood group antigen. Central to this interaction is a salt bridge between Glu658 in Band 3 and Arg61 in Glycophorin A (Bruce et al., 1995). Wr(a^+^b^+^) heterozygotes also have Lys at residue 658. Glycophorin A interacts with Band 3 in the ER facilitating its trafficking to the cell surface (Groves and Tanner, 1992; Pang and Reithmeier, 2009). MD simulations of the membrane domain of Band 3 and the dimeric TM portion of Glycophorin A were carried out in a complex lipid bilayer (Kalli and Reithmeier, 2018). Glycophorin A was positioned in the greatest distance from Band 3 while maintaining the Glu658-Arg61 ionic interaction. Glycophorin A moved closer to Band 3 suggesting that in addition to the interaction between Glu658 and Arg61 there are also interactions between the helical TM segments and extracellular regions of Glycophorin A and similar regions in Band 3 (Figure 4A). Figure 4B shows the residues involved in the interaction of Band 3 and Glycophorin A. Changing Glu658 to Lys resulted in loss of the interaction with Arg61 but the TM region of Glycophorin A still interacted with Band 3. This suggests that the Wright blood group antigen is probably due to a local conformational change in the epitope rather than complete dissociation of the Glycophorin A/Band 3 complex. Large-scale simulations showed that the Glycophorin A dimer can bridge Band 3 dimers resulting in the dynamic formation of a supramolecular complex of long strands of alternating Band 3 and Glycophorin A dimers. It would be of considerable interest to use MD simulations to examine the effect of Band 3 mutations (e.g., G701D) on the structure of Band 3 and its interaction with Glycophorin A.

## Band 3-Lipid Interactions

**Figure 5:**
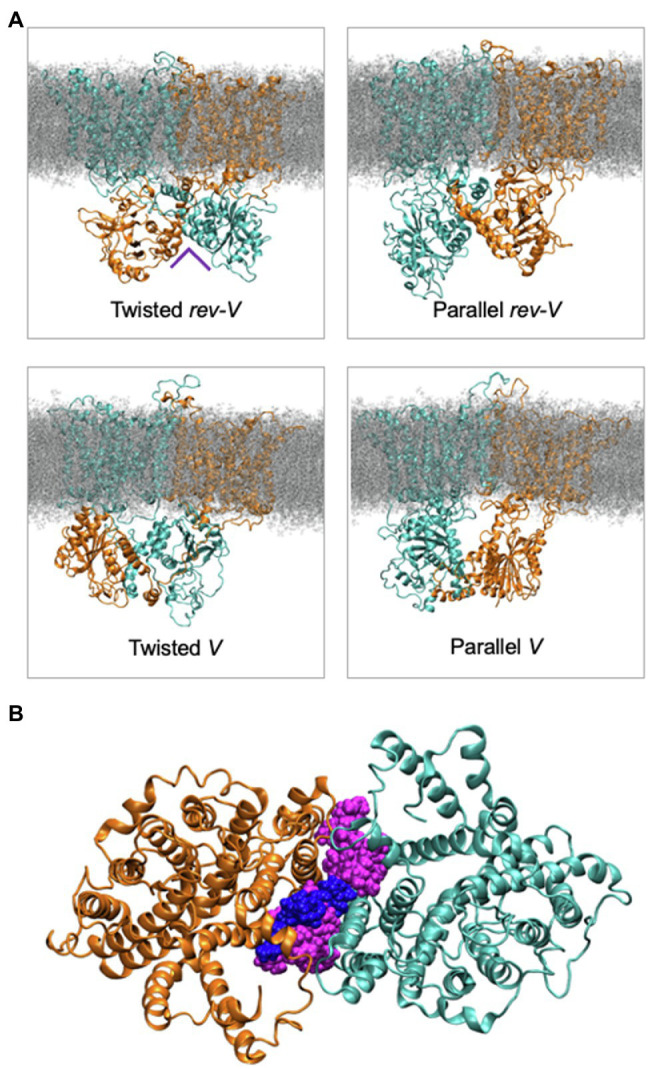
**(A)** Molecular dynamics simulation snapshots showing the four different models in the relative orientation of the cytoplasmic domain in the intact Band 3 in a complex lipid bilayer. One of Band 3 monomers is shown in orange and the other in cyan. Lipids are shown in grey. The Twisted models (left) indicate a domain swap of the membrane and cytoplasmic domains. **(B)** Cytoplasmic view of a simulation with the intact Band 3 highlighting the lipids at the dimer interface during the simulations. The cytosolic region of Band 3 and the rest of the bilayer are omitted for clarity (Adapted from de Vecchis et al., 2019).

Band 3 is embedded in a complex lipid bilayer and has extensive interactions with both phospholipids and cholesterol. MD simulations of the membrane domain Band 3 have revealed specific binding sites for acidic lipids and cholesterol (Kalli and Reithmeier, 2018). Cholesterol was found at the dimer interface where it may play a structural role in stabilizing the interaction between the gate domains of the two subunits (Figure 5B). In addition, the MD simulation studies have also suggested that there is enough space in the dimer interface for one phospholipid or sphingomyelin molecule. Cholesterol is known to inhibit anion transport and so may also play a regulatory role (Gregg and Reithmeier, 1983). Although Band 3 interacted with both 1-palmitoyl-2-oleoyl-phosphatidylserine (POPS) and phosphatidylinositol 4,5-bisphosphate (PIP_2_) lipids, MD simulations have shown a strong preference for PIP_2_ lipids. PIP_2_ provides binding sites for cytoplasmic proteins such as Protein 4.1. Band 3 is therefore a central organization center in the red blood cell membrane by direct interaction with proteins and associated lipids like PIP_2_. Thus, the interaction of proteins with Band 3 may be regulated *via* modification of associated lipids.

Molecular dynamics simulations and modeling of intact Band 3 were used to provide an atomistic view of the interaction and dynamics of the cytoplasmic domain (missing the first 54 disordered residues), the membrane domain, and the connecting linker in a model of AE1 in a complex lipid bilayer (de Vecchis et al., 2019). During the simulations, the cytoplasmic domain moved closer to the membrane domain forming a more compact structure. The flexible linker was involved in the interaction of the membrane and cytoplasmic region but its removal after Band 3 adopted a more compact structure did not change the stability of the structure. Analysis of four possible orientations of the cytoplasmic region relative to the membrane region of Band 3 based on available experimental data indicated that a structure with the C-terminal dimerization arms (residues 314–347) of the cytoplasmic region facing the transmembrane region of Band 3 was most likely (Figure 5). The two twisted models in which there was a domain swap in the orientation of the cytoplasmic domain allowed closer packing of the complex compared to the other orientations. Structures of SLC26 transporters, which also feature a 14 TM 7 + 7 inverted repeat topology (Baranovski et al., 2020), revealed a domain swap of the C-terminal STAS domain with major interactions between the STAS domain and the membrane domain being responsible for dimer stability (Chang et al., 2019; Walter et al., 2019). There are interactions of the cytoplasmic domain of Band 3 with the membrane domain in a cross-over structure that may stabilize the dimer; however, both domains in isolation remain dimeric. This structural and functional independence of the two domains in Band 3 may not be a feature of other members of the SLC4 family, whose transport activity is regulated by intracellular pH and other factors as opposed to Band 3 that works near maximum velocity (Zhang et al., 1996).

The red blood cell membrane contains lipid nanodomains (“rafts”) enriched in cholesterol and sphingomyelin (Simons and Ikonen, 1997). Atomistic MD simulations with the membrane region of Band 3 inserted in membranes that contained 1,2-dipalmitoyl-phosphatidylcholine (DPPC), 1,2-dilinoleoyl-phosphatidylcholine (DLiPC), and cholesterol (4:3:3) showed that there was an asymmetric interaction of Band 3 with the phase-separated lipid nano domains in the two leaflets in this bilayer (Jin et al., 2020). At the end of these simulations, the contacts between Band 3 and DLiPC and DPPC were ~4:1 in the outer leaflet and ~3:2 in the inner leaflet. This suggests that in the outer leaflet the protein contacts mainly low-Tm Ld domains, whilst in the inner leaflet Band 3 contacts both Ld and high-Tm lipid and cholesterol Lo domains. Simulations of Band 3 in a DPPC, DLiPC with 30 or 50 mol % cholesterol showed that a higher cholesterol concentration results in the deformation of some helices in Band 3. Simulation with a bilayer that contained a 1,2-distearoyl-phosphatidylserine (DSPS), DLiPC, and cholesterol revealed strong electrostatic interactions between phosphatidylserine (PS) lipids and Band 3. As a result, Band 3 interacts with Lo domains at specific sites in both leaflets; this is not the case in the simulations with DPPC, DLiPC, and cholesterol.

The bacterial purine transport UraA has a similar 7 + 7 inverted topology as Band 3 (Lu et al., 2011). MD simulations of the UraA monomer in a complex lipid bilayer revealed specific interactions with lipids, particularly cardiolipin (Kalli et al., 2015). MD simulations of the fungal proton-driven purine transporter UapA support the essential role of lipids in stabilizing its functional dimeric structure and an elevator model for transport (Alguel et al., 2016). MD simulations of a mutant form of UapA (G411V) trapped in the inward-facing state identified an essential arginine-rich lipid-binding site at the dimer interface (Pyle et al., 2018). The fumarate transporter SLC26Dg crystallized as a monomer but is reported to be a dimer in a lipid bilayer (Geertsma et al., 2015). All of these studies point to an essential role of lipids in stabilizing the dimeric state of these transport proteins.

## Conformational Changes Associated With Transport

Band 3, like many facilitated transporters, works by an alternating access model. The crystal structure of the membrane domain has revealed key features of this mode of transport (Reithmeier et al., 2016). First, there is a single anion binding site located between the N-termini of two short α-helices (TM3 and 10) with participation of the side chain of Arg730, consistent with earlier kinetic studies. This site is occupied by one of the sulfonate groups of the inhibitor Diisothiocyanostilbenedisulfonate (DIDS), which locked the protein into the outward-facing state. A second feature of Band 3 is the presence of two domains, a so-called gate domain at the dimer interface and a core domain that has a predominate interaction with the lipid bilayer. The structure resembles a mobile bone and socket with slippery hydrophobic interacting surfaces. The interface between these two domains is dynamic allowing alternating substrate access from the outside and inside.

Molecular dynamics simulations combined with experimental data have shown two substrate binding sites in the outward facing state of Band 3 and the related human electrogenic sodium bicarbonate cotransporter (HNBCe1, SLC4A4; Zhekova et al., 2021). One site is located at the entry of the outward facing cavity and the other one in a more central position toward the middle of the protein. Band 3 residue R730 was shown to be critical for anion binding for both the entry and central binding sites. The cryo-electron microscopic structure of a sodium-dependent chloride/bicarbonate exchanger (SLC4A8) in the outward-facing state had well-defined densities for both the Na^+^ close to D800 and bicarbonate in the central substrate binding site (Wang et al., 2021). Interestingly, the dimeric structure was further stabilized by a compact extracellular N-glycosylated domain between TM5 and 6 that contained two disulfide bonds. Dimerization would confer stability between the two interacting gate domains in the plane of the membrane, suggesting that it is the core domain that moves during the transport cycle.

## Rocker and Elevator Transport Models

There are two basic structural mechanisms for alternating access model of membrane transport: the rocker switch or rocking bundle model and the elevator model (Diallinas, 2021). In both models, there is a central substrate binding site located about half-way across the membrane. The substrate binding sites are engineered through evolution to enable specificity for substrates. Conformational changes allow alternating access of the substrate binding site to one side of the membrane and then the other. There is no open passageway or channel that would allow leakage of substrate across the membrane. In the case of Band 3, it may be Glu681 that occupies the empty anion binding site, thereby preventing the conformational change associated with transport.

**Figure 6:**
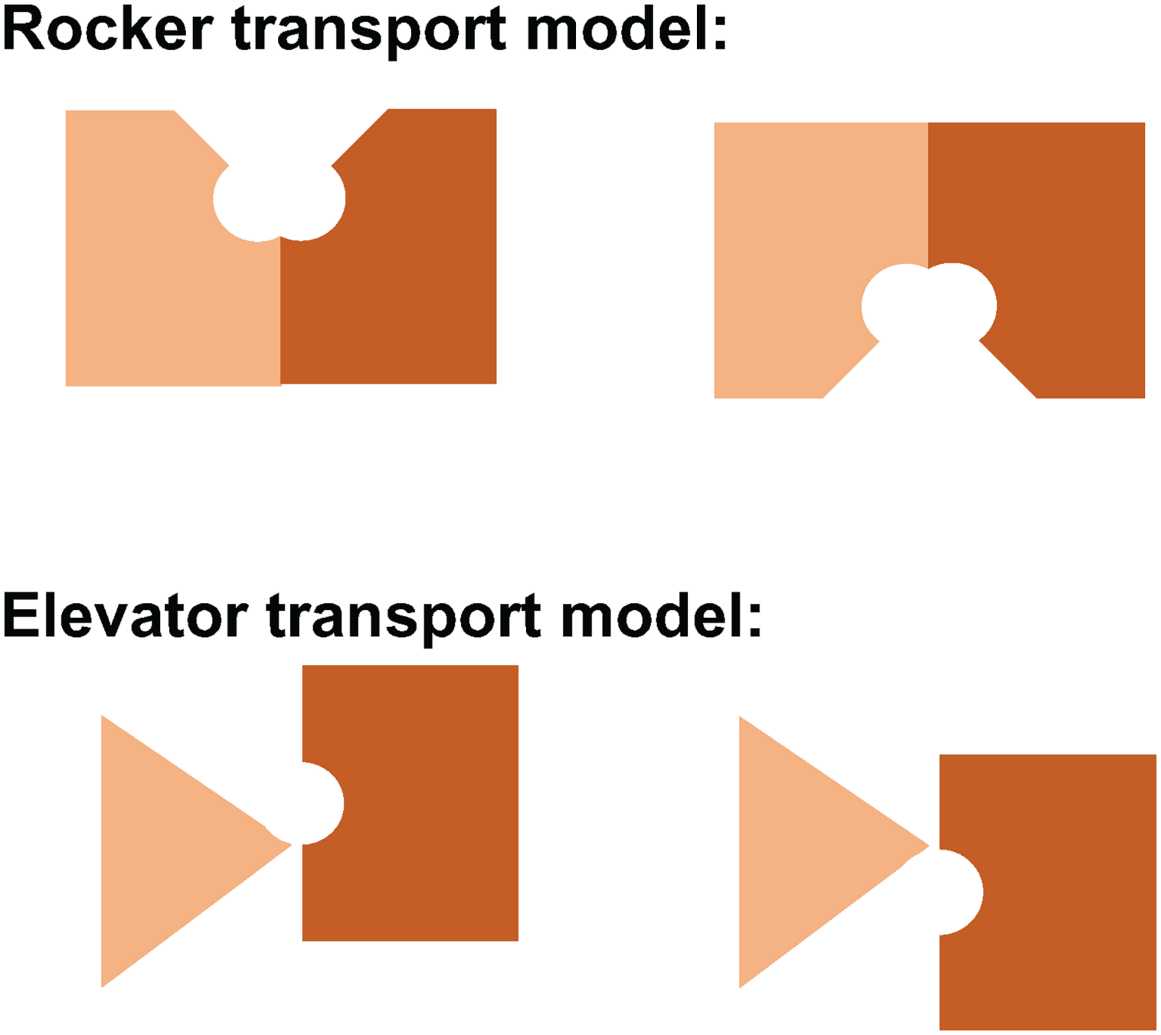
Schematic representation of the rocker and elevator models of transport. In the rocker model (**above**), both domains undergo major conformational changes to allow alternating access of the central substrate binding site to one side of the membrane or the other. In the elevator model (**below**), the stationary domain (triangle) remains fixed in the plane of the membrane while the mobile domain (rectangle) moves up or down to allow alternating access to the central substrate binding site. In Band 3, the gate domain at the dimer interface is immobile while the core domain that contains the central substrate binding site moves relative to the plane of the membrane.

The rocker model (Figure 6) typically involves the movement of two symmetrical domains of the membrane protein that both contribute to substrate binding, typical for the 6 + 6 parallel two domain structure common to the major facilitated transporter superfamily (Drew et al., 2021). Indeed, the conformational changes associated with rocker mechanism of transport can occur without substrate in cases like the glucose transporter, returning the empty carrier to an outward-facing state to pick up another substrate. In the rocker model, there is no significant movement of the substrate-binding site relative to the membrane.

In contrast, the elevator model (Figure 6) features a stationary domain and a mobile substrate binding domain. The stationary domain is created by stable interactions between subunits at the dimer interface. It is the substrate binding domain that moves relative to the membrane. In Band 3, the stationary domain is the gate domain and the mobile domain is the core domain. It is the binding of substrates that permits the conformational changes associated with transport in Band 3 since the empty carrier cannot undergo the conformational change associated with transport. Simply put, the elevator cannot operate without a passenger substrate. It is unlikely that simple rigid body movements of two domains fully capture the complexity of the conformational changes associated with transport that allows binding and release of substrates. MD simulations may provide insights into the full extent of these structural changes.

The 7 + 7 TM inverted repeats consisting of TM1-7 and TM8-14 in each Band 3 monomer have a similar fold. The 7 + 7 inverted repeat structure is consistent with an alternating access model of transport. The repeats are however not homologous and differ greatly in amino acid sequence resulting in an asymmetric structure. In addition, it is not the repeats that move relative to one another but rather the core and gate domains.

**Figure 7:**
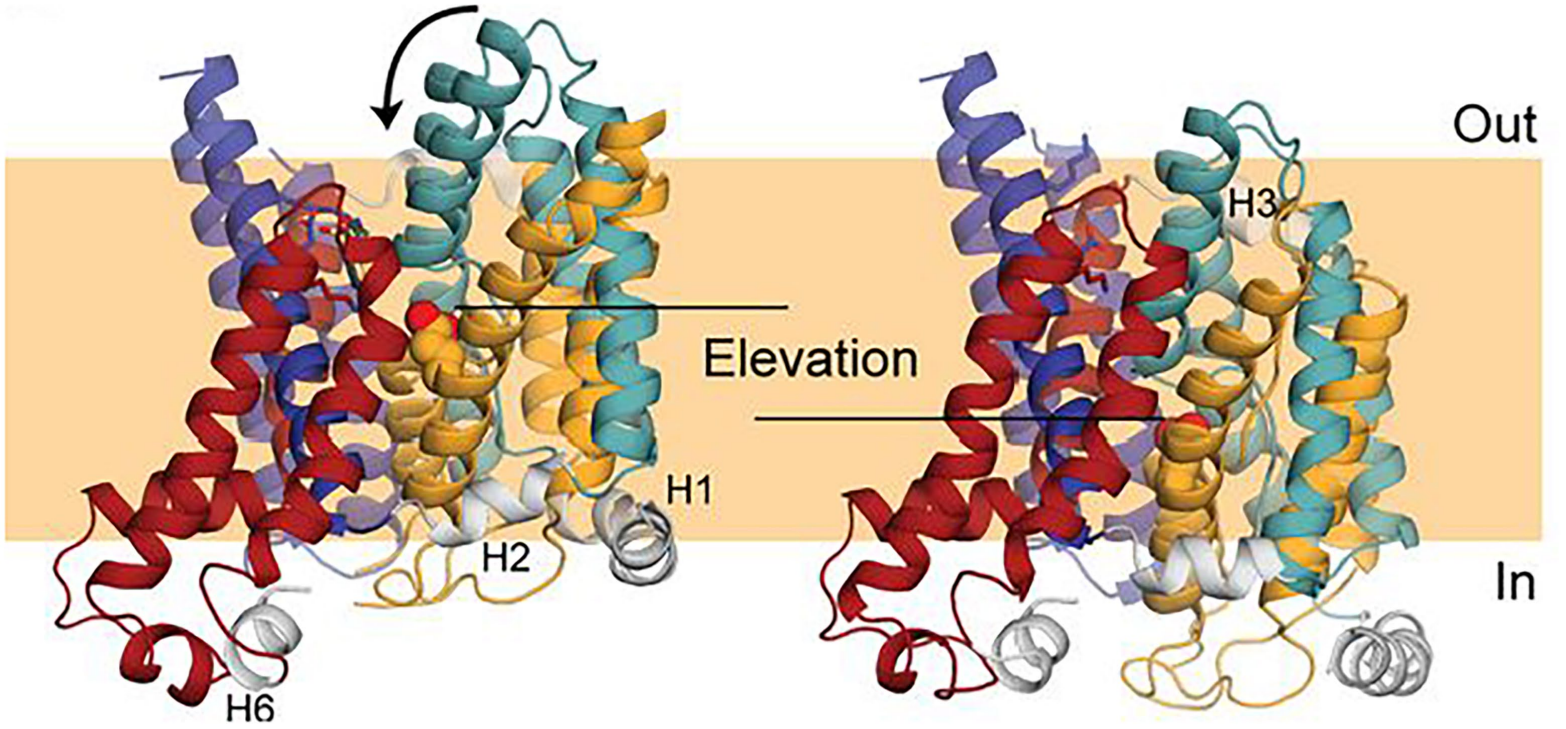
Comparison of a repeat-swap model of the inward-facing model of Band 3 (**right**) with the 4YZF outward-facing crystal structure (**left**). The difference between the two states suggested an elevator-like conformational change with movement of the core domain relative the fixed gate domain. This figure was taken from Ficici et al. (2017) with permission of the corresponding authors.

A model (Figure 7) of the inward-facing state of Band 3 was constructed using repeat-swap homology modeling whereby the second seven TM repeat assumes the conformation of the first seven TM repeat and vice versa (Ficici et al., 2017). The modeling suggested that the transport mechanism of Band 3 involves an elevator-like translation of about 8 Å to the bilayer normal (~11 Å in total) and rotation of ~17 degrees of the substrate-binding core domain relative to the nearly stationary dimerization gate domain. In the outward facing state, the water-filled passage is lined by TM1 and 3 from the core domain and TM5 and 13 from the gate domain, while in the inward-facing state the 7 + 7 symmetry-related TM8 and 10 and TM6 and 12 are predicted to line the passage. Early studies showed that crosslinking the two monomers *via* residues now known to be within the gate domain in the TM5-6 loop (Lys551–Lys562) did not inhibit transport indicating that the dimer interface does not undergo a major conformational change during transport (Jennings and Nicknish, 1985).

Support for the elevator model for Band 3 comes from structural studies of the related plant (Thurtle-Schmidt and Stroud, 2016) and yeast SLC4 borate transporter BorA (Coudray et al., 2017). The Arapidopsis protein crystallized as a dimer with each subunit having the same 14 TM fold as Band 3. Although no substrate could be identified at the 4.1 Å resolution, the structure was in an occluded state in which the core domains are rotated inward toward the gate domain with the extracellular ends of TM1, 3 and 8 moving inward by about 8 Å to close off the extracellular side of the protein. The structure of the yeast BorA protein was determined from helical membrane crystals at 6 Å resolution. Homology modeling (~23% sequence identity) and MD simulations using Band 3 as a template and water accessibility revealed that the BorA was in the inward-facing conformation with a rigid body rotation of the core domain by ~10 degrees against the immobile gate domain relative to Band 3. Comparison of the inward-facing structure with an outward-facing model of BorA did not reveal any translation of the core domain, leading the author to suggest that BorA does not operate by an elevator mechanism. It is important to recognize that the structure represents a substrate-bound occluded form of BorA and not the empty carrier in its full inward-facing state. The same can be said for the structure of Band 3 that was determined in the presence of the bound inhibitor DIDS and does not represent the empty outward-facing state. Thus, the conformational changes associated with the full transport cycle may be more profound that observed in substrate or inhibitor-bound forms.

The 7 + 7 inverted repeat topology of the SLC4 family is also shared with members of the SLC23 family of nucleobase transporters. The crystal structure of the proton-driven uracil symporter, UraA in an inward-facing state was the first to reveal the 14 TM 7 + 7 inverted repeat topology (Lu et al., 2011). The UraA structure was originally reported as a monomer; however, the crystal lattice indicated that the protein is dimer with interactions between the gate domains. The structure of UraA with bound uracil in an occluded state in dimeric form was subsequently determined (Yu et al., 2017). The core domains of the inward-facing and occluded state could be superimposed, while there were conformational changes within the gate domain. There are structural changes in TM5 and its inverted partner TM 12. Both TM5 and 12 rotate around an axis perpendicular to the interface between the core and gate domains. TM5 and 12 are both straight α-helices in the inward-facing state but both are kinked in the occluded state. The periplasmic end of TM5 moves toward the core domain, while the cytoplasmic end of TM12 moves away creating an inward-facing opening. In addition, the periplasmic loop connecting TM13 and 14 formed a rigid β-hairpin “paddle” in the occluded state. The binding of uracil in the core domain remains the same in both states. There is a translocation of ~5 Å of the substrate in the core domain between the occluded state of UraA and inward-facing state of UapA, consistent with an elevator model of transport. There were also significant conformational changes observed in the gate domain of UraA. The higher resolution of the occluded state at 2.5 Å identified water molecules bridging uracil to essential water-binding residues (Wat 1 to Ser72 and Glu241 and Wat 2 to Glu241 and His245). In the previous inward-facing state, a detergent molecule occupied the position of the Wat2 water molecule. MD simulations of UraA in a lipid bilayer identified a proton translocation pathway involving loss of water binding to Glu241 and interaction with His245.

UraA exists as a functional dimer in the membrane and is equilibrium between monomer and dimer in detergent (Yu et al., 2017). This points once again to the essential role that lipids play in the stabilization of the oligomeric state of membrane transport proteins. The dimer is held together by hydrophobic interactions between the gate domains with TM13 in one subunit packed into TM5 and 12 in the other subunit.

Our MD simulations of monomeric UraA in the absence of uracil in a lipid bilayer mimicking the bacterial inner membrane found that cardiolipin interacted with three specific sites, one on the outer surface (Arg321) and two on the inner surface (Arg299/Arg4 and Arg265/Lys109; Kalli et al., 2015). Mutation of the interacting arginine or lysine residues at these sites resulted loss of lipid binding. Cardiolipin may stabilize the structure of UraA and promote dimer formation. The cardiolipin on the outer surface of UraA is close to the transport pathway and may act as a “proton trap” to channel protons to the transporter. Cardiolipin on the inner surface may channel protons from the transporter. Cardiolipin may play a similar functional role in proton translocation in bacterial and mitochondrial proton-driven symporters that are embedded in membrane rich in cardiolipin. In the absence of uracil, the MD simulations of the UraA monomer showed that the inward-facing transporter assumed a more closed state, due to relative movement of the gate and core domains. This closed state in the absence of substrate may represent an intermediate in the empty carrier changing from the inward-facing to outward-facing state to complete the transport cycle. The transport cycle involves sequential binding of the proton and uracil to the outward-facing state, a conformational change through an occluded state to the inward facing state, sequential release of the proton and uracil, and finally a conformational change of the now empty carrier to return to the original outward-facing state. This transport cycle may explain why symporters (co-transporters) tend to be crystallized in the inward-facing state in the presence of substrate. In contrast, antiporters (exchangers) undergo the conformational change in the presence of substrate and must be trapped in one conformational state with the use of inhibitors.

The structure of the proton-driven nucleobase xanthine symporter, UapA from Aspergillus was determined in the substrate-bound inward-facing state (Alguel et al., 2016). UapA had the same 7 + 7 inverted repeat fold as UraA with corresponding core and gate domains. The substrate xanthine was held within the core domain between the two TM3 and 10 half helices in a similar position as uracil in UraA. UapA crystallized as a dimer with extensive interactions involving TMs12-14 in the gate domain. The dimeric structure of UapA was found to be essential for trafficking to the cell surface and transport function (Kourkoulou et al., 2019). Mutations affecting transport and substrate specificity were found in the substrate-binding core domain as expected (e.g., Phe 406) but also in the dimerization gate domain in TM 12 and 14. A disulfide bond in the extracellular loop connecting TM 3 and 4 was essential for proper folding and trafficking. Mutation of Arg481 affects substrate specificity. This residue is located close to the substrate binding site of its partner subunit. Co-expression of inactive mutants with the normal protein resulted in a dominant-negative effect on transport consistent with the dimer being the functional transport unit. A model of the outward-facing state of UapA was built using Band 3 as a template. In UapA, the core domain is displaced downward toward by ~10 Å in the inward-facing state, while the gate domain remains fixed in position in the membrane. Tightly-bound lipids (PI and PE) were found associated with UapA and stabilized the dimeric form of the protein (Pyle et al., 2018). MD simulations localized PI to specific basic residues on the cytoplasmic surface near the dimer interface. Mutation of specific arginine residues involved in PI binding resulted in destabilization of the dimer and loss of transport activity.

There are likely subtle but important differences in the details of the conformational changes in the 14 TM 7 + 7 inverted repeat transporters. For example, in the UraA proton-uracil co-transporter (symporter), the empty carrier must be able to move from the inward-facing to the outward-facing state to complete the transport cycle. In exchangers (antiporters) like Band 3, the empty carrier cannot undergo this conformational change to the outward state without bound substrate.

The crystal structure of the membrane domain of Band 3 is locked in the outward-facing state and we do not yet have a structure of the inward-facing conformation. We are currently using the inward-facing and occluded states of UraA to build equivalent models of Band 3. It is unlikely that transport occurs by simple rigid body movement of the two domains. MD simulations will allow us to examine the full extent of the structural changes associated with the transition from outward to inward-facing state of Band 3 with a focus on the relative movement of the gate and core domains.

## Senescence: An Example of Conformational Dynamics in Band 3

Finally, an excellent example of the conformational dynamics of Band 3 is the senescence antigen (Badior and Casey, 2021). Early work showed that the Band 3 sequence encompassing residues 812–830 plays a role in binding endogenous senescence antibodies to aged red blood cells. In the crystal structure of the membrane domain, this region is on the cytoplasmic side of the membrane and consists of two short α-helices joined by a reverse turn linking TM12 and 13 (Figure 3). Using cysteine scanning mutagenesis of Band 3 and the membrane domain expressed in HEK cells, it was demonstrated that this region can assume an extracellular disposition. TM13 and 14 are shorter than typical TM α-helices and may not have a very stable interaction with the lipid bilayer (Deber et al., 2002). The interface region of the bilayer is about 15 Å thick allowing interacting TM segments of membrane proteins some flexibility in their position relative to the plane of the membrane (Reithmeier, 1995). The ~30 Å thick hydrophobic zone is likely a greasy slide, again allowing some movement of mobile domains. Cys843 has been identified as a site of palmitoylation in Band 3, yet is located in the middle of TM13. Fatty acylated residues are typically located at the membrane interface with the fatty acid positioned along with other fatty acyl groups in the lipid bilayer (Cheung and Reithmeier, 2004). This indicates that TM13 has a dynamic aspect and that the non-enzymatic palmitoylation of Cys843 may occur as red blood cells age and be part of the senescence clock.

## Conclusion

Even after more than 50 years of study, key knowledge gaps in our understanding of the structure and function of Band 3 remain. We still do not have experimentally-derived structures of intact Band 3 or other members of the human SLC4 family, the inward-facing conformation, the tetramer, or complexes with cytoskeletal proteins like ankyrin. The nature of the conformational changes associated with transport is not fully described, and the effect of mutations linked to disease such as SAO on protein folding requires further study. Many of these remaining questions can be addressed by MD simulations, which can provide insights to an atomistic level. In addition, cryo-electron microscopy holds considerable promise to reveal the structure of Band 3 complexes, as well as mutant forms of Band 3 expressed in yeast (Sekler et al., 1995) or transfected cells (Okawa et al., 2014).

## Author Contributions

RAFR and ACK contributed equally to the writing of this review. References were complied by RAFR, and figures were prepared by ACK. All authors contributed to the article and approved the submitted version.

## Funding

RAFR was supported by funding from the Temerty Faculty of Medicine, University of Toronto.

## Conflict of Interest

The authors declare that the research was conducted in the absence of any commercial or financial relationships that could be construed as a potential conflict of interest.

## Publisher’s Note

All claims expressed in this article are solely those of the authors and do not necessarily represent those of their affiliated organizations, or those of the publisher, the editors and the reviewers. Any product that may be evaluated in this article, or claim that may be made by its manufacturer, is not guaranteed or endorsed by the publisher.

## References

[ref1] Alguel Y. Amillis S. Leung J. Lambrinidis G. Capaldi S. Scull N. J.. (2016). Structure of eukaryotic purine/H+ symporter UapA suggests a role for homodimerization in transport activity. Nat. Commun. 7:11336. doi: 10.1038/ncomms11336, PMID: 27088252 PMC4837479

[ref2] Alper S. L. (2009). Molecular physiology and genetics of Na^+^-independent SLC4 anion exchangers. J. Exp. Biol. 212, 1672–1683. doi: 10.1242/jeb.029454, PMID: 19448077 PMC2683012

[ref3] Arakawa T. Kobayashi-Yurugi T. Alguel Y. Iwanari H. Hatae H. Iwata M.. (2015). Crystal structure of the anion exchanger domain of human erythrocyte band 3. Science 350, 680–684. doi: 10.1126/science.aaa4335, PMID: 26542571

[ref4] Badior K. E. Casey J. R. (2021). Large conformational dynamics in band 3 protein: significance for erythrocyte senescence signalling. Biochim. Biophys. Acta Biomembr. 1863:183678. doi: 10.1016/j.bbamem.2021.183678, PMID: 34175296

[ref5] Baranovski B. M. Fremder M. Ohana E. (2020). “Properties, structure, and function of the solute carrier 26 family of anion transporters,” in Studies of Epithelial Transporters and Ion Channels: Ion Channels and Transporters of Epithelia in Health and Disease (Physiology in Health and Disease). *Vol*. 3. eds. Hamilton K. L. Devor D. C. (Springer), 467–493.

[ref6] Bruce L. J. Beckmann R. Ribeiro M. L. Peters L. L. Chasis J. A. Delaunay J.. (2003). A band 3-based macrocomplex of integral and peripheral proteins in the RBC membrane. Blood 101, 4180–4188. doi: 10.1182/blood-2002-09-28242002-09-2824, PMID: 12531814

[ref7] Bruce L. J. Ring S. M. Anstee D. J. Reid M. E. Wilkinson S. Tanner M. J. (1995). Changes in the blood group Wright antigens are associated with a mutation at amino acid 658 in human erythrocyte band 3: a site of interaction between band 3 and glycophorin A under certain conditions. Blood 85, 541–547. doi: 10.1182/blood.V85.2.541.541, PMID: 7812009

[ref8] Casey J. R. Reithmeier R. A. F. (1991). Analysis of the oligomeric state of band 3, the anion transport protein of the human erythrocyte membrane, by size exclusion high performance liquid chromatography: oligomeric stability and origin of heterogeneity. J. Biol. Chem. 266, 15726–15737. doi: 10.1016/S0021-9258(18)98470-X, PMID: 1874731

[ref9] Chambers E. J. Bloomberg G. B. Ring S. M. Tanner M. J. (1999). Structural studies on the effects of the deletion in the red cell anion exchanger (band 3, AE1) associated with south east Asian ovalocytosis. J. Mol. Biol. 285, 1289–1307. doi: 10.1006/jmbi.1998.2392, PMID: 9887277

[ref10] Chang Y. N. Geertsma E. R. (2017). The novel class of seven transmembrane segment inverted repeat carriers. Biol. Chem. 398, 165–174. doi: 10.1515/hsz-2016-0254, PMID: 27865089

[ref11] Chang Y. N. Jaumann E. A. Reichel K. Hartmann J. Oliver D. Hummer G.. (2019). Structural basis for functional interactions in dimers of SLC26 transporters. Nat. Commun. 10:2032. doi: 10.1038/s41467-019-10001-w, PMID: 31048734 PMC6497670

[ref12] Cheung J. C. Cordat E. Reithmeier R. A. (2005). Trafficking defects of the southeast Asian ovalocytosis deletion mutant of anion exchanger 1 membrane proteins. Biochem. J. 392, 425–434. doi: 10.1042/BJ20051076, PMID: 16107207 PMC1316280

[ref13] Cheung J. C. Reithmeier R. A. F. (2004). Palmitoylation is not required for trafficking of human anion exchanger 1 to the cell surface. Biochem. J. 378, 1015–1021. doi: 10.1042/bj20030847, PMID: 14640982 PMC1224004

[ref14] Cheung J. C. Reithmeier R. A. F. (2005). Membrane integration and topology of the first transmembrane segment in normal and southeast Asian ovalocytosis human erythrocyte anion exchanger 1. Mol. Membr. Biol. 22, 203–214. doi: 10.1080/09687860500093115, PMID: 16096263

[ref15] Cordat E. Reithmeier R. A. F. (2014). Structure, function, and trafficking of SLC4 and SLC26 anion transporters. Curr. Top. Membr. 73, 1–67. doi: 10.1016/B978-0-12-800223-0.00001-3, PMID: 24745980

[ref16] Coudray N. Seyler S. Lasala R. Zhang Z. Clark K. M. Dumont M. E.. (2017). Structure of the SLC4 transporter Bor1p in an inward-facing conformation. Protein Sci. 26, 130–145. doi: 10.1002/pro.3061, PMID: 27717063 PMC5192975

[ref17] de Vecchis D. Reithmeier R. A. F. Kalli A. C. (2019). Molecular simulations of intact anion exchanger 1 reveal specific domain and lipid interactions. Biophys. J. 117, 1364–1379. doi: 10.1016/j.bpj.2019.08.029, PMID: 31540709 PMC6818359

[ref18] Deber C. M. Liu L.-P. Wang C. Goto N. K. Reithmeier R. A. R. (2002). The hydrophobicity threshold for peptide insertion into membranes. ChemInform 52, 465–479. doi: 10.1016/S1063-5823(02)52018-4

[ref19] Diallinas G. (2021). Transporter specificity: a tale of loosened elevator-sliding. Trends Biochem. Sci. 46, 708–717. doi: 10.1016/j.tibs.2021.03.007, PMID: 33903007

[ref20] Drew D. North R. A. Nagarathinam K. Tanabe M. (2021). Structures and general transport mechanisms by the major facilitator superfamily (MFS). Chem. Rev. 121, 5289–5335. doi: 10.1021/acs.chemrev.0c00983, PMID: 33886296 PMC8154325

[ref21] Falke J. J. Chan S. I. (1985). Evidence that anion transport by band 3 proceeds via a ping-pong mechanism involving a single transport site. A 35 cl NMR study. J. Biol. Chem. 260, 9537–9544. doi: 10.1016/S0021-9258(17)39268-2 4019484

[ref22] Ficici E. Faraldo-Gómez J. D. Jennings M. L. Forrest L. R. (2017). Asymmetry of inverted-topology repeats in the AE1 anion exchanger suggests an elevator-like mechanism. J. Gen. Physiol. 149, 1149–1164. doi: 10.1085/jgp.201711836, PMID: 29167180 PMC5715908

[ref23] Fowler P. W. Sansom M. S. P. Reithmeier R. A. F. (2017). Effect of the southeast Asian Ovalocytosis deletion on the conformational dynamics of signal-anchor transmembrane segment 1 of red cell anion exchanger 1 (AE1, band 3, or SLC4A1). Biochemistry 56, 712–722. doi: 10.1021/acs.biochem.6b00966, PMID: 28068080 PMC5299548

[ref24] Geertsma E. R. Chang Y. N. Shaik F. R. Neldner Y. Pardon E. Steyaert J.. (2015). Structure of a prokaryotic fumarate transporter reveals the architecture of the SLC26 family. Nat. Struct. Mol. Biol. 22, 803–808. doi: 10.1038/nsmb.3091, PMID: 26367249

[ref25] Gregg V. A. Reithmeier R. A. F. (1983). Effect of cholesterol on phosphate uptake by human red blood cells. FEBS Lett. 157, 159–164. doi: 10.1016/0014-5793(83)81137-5, PMID: 6862013

[ref26] Groves J. D. Tanner M. J. (1992). Glycophorin A facilitates the expression of human band 3-mediated anion transport in Xenopus oocytes. J. Biol. Chem. 267, 22163–22170. doi: 10.1016/S0021-9258(18)41649-3, PMID: 1385395

[ref27] Groves J. D. Wang L. Tanner M. J. (1998). Functional reassembly of the anion transport domain of human red cell band 3 (AE1) from multiple and non-complementary fragments. FEBS Lett. 433, 223–227. doi: 10.1016/s0014-5793(98)00909-0, PMID: 9744799

[ref28] Gupta K. Donlan J. A. C. Hopper J. T. S. Uzdavinys P. Landreh M. Struwe W. B.. (2017). The role of interfacial lipids in stabilizing membrane protein oligomers. Nature 541, 421–424. doi: 10.1038/nature20820, PMID: 28077870 PMC5501331

[ref29] Hunte C. Richers S. (2008). Lipids and membrane protein structures. Curr. Opin. Struct. Biol. 18, 406–411. doi: 10.1016/j.sbi.2008.03.008, PMID: 18495472

[ref30] Jennings M. L. (2021). Cell physiology and molecular mechanism of anion transport by erythrocyte band 3/AE1. Am. J. Physiol. Cell Physiol. 321, C1028–C1059. doi: 10.1152/ajpcell.00275.2021, PMID: 34669510 PMC8714990

[ref31] Jennings M. L. Nicknish J. S. (1985). Localization of a site of intermolecular cross-linking in human red blood cell band 3 protein. J. Biol. Chem. 260, 5472–5479. doi: 10.1016/S0021-9258(18)89046-9, PMID: 3988763

[ref32] Jin Y. Liang Q. Tieleman D. P. (2020). Interactions between band 3 anion exchanger and lipid nanodomains in ternary lipid bilayers: atomistic simulations. J. Phys. Chem. B 124, 3054–3064. doi: 10.1021/acs.jpcb.0c01055, PMID: 32216275

[ref33] Kalli A. C. Reithmeier R. A. F. (2018). Interaction of the human erythrocyte band 3 anion exchanger 1 (AE1, SLC4A1) with lipids and glycophorin A: molecular organization of the Wright (Wr) blood group antigen. PLoS Comput. Biol. 14:e1006284. doi: 10.1371/journal.pcbi.1006284, PMID: 30011272 PMC6080803

[ref34] Kalli A. C. Sansom M. S. P. Reithmeier R. A. F. (2015). Molecular dynamics simulations of the bacterial UraA H<sup>+</sup>-uracil symporter in lipid bilayers reveal a closed state and a selective interaction with cardiolipin. PLoS Comput. Biol. 11:e1004123. doi: 10.1371/journal.pcbi.1004123, PMID: 25729859 PMC4346270

[ref35] Knauf P. A. (1979). Erythrocyte anion exchange and the band 3 protein: transport kinetics and molecular structure. Curr. Top. Membr. Transp. 12, 249–363. doi: 10.1016/S0070-2161(08)60259-2

[ref36] Kourkoulou A. Grevias P. Lambrinidis G. Pyle E. Dionysopoulou M. Politis A.. (2019). Specific residues in a purine transporter are critical for dimerization, ER exit, and function. Genetics 213, 1357–1372. doi: 10.1534/genetics.119.302566, PMID: 31611232 PMC6893392

[ref37] Low P. S. (1986). Structure and function of the cytoplasmic domain of band 3: center of erythrocyte membrane-peripheral protein interactions. Biochim. Biophys. Acta 864, 145–167. doi: 10.1016/0304-4157(86)90009-2, PMID: 2943319

[ref38] Lu F. Li S. Jiang Y. Jiang J. Fan H. Lu G.. (2011). Structure and mechanism of the uracil transporter UraA. Nature 472, 243–246. doi: 10.1038/nature09885, PMID: 21423164

[ref39] Lux S. E. John K. M. Kopito R. R. Lodish H. F. (1989). Cloning and characterization of band 3, the human erythrocyte anion-exchange protein (AE1). Proc. Natl. Acad. Sci. U. S. A. 86, 9089–9093. doi: 10.1073/pnas.86.23.9089, PMID: 2594752 PMC298439

[ref40] Macara I. G. Cantley L. C. (1981). Interactions between transport inhibitors at the anion binding sites of the band 3 dimer. Biochemistry 20, 5095–5105. doi: 10.1021/bi00521a001, PMID: 7295667

[ref41] Maneri L. R. Low P. S. (1989). Fatty acid composition of lipids which copurify with band 3. Biochem. Biophys. Res. Commun. 159, 1012–1019. doi: 10.1016/0006-291x(89)92209-2, PMID: 2930548

[ref42] Mankelow T. J. Satchwell T. J. Burton N. M. (2012). Refined views of multi-protein complexes in the erythrocyte membrane. Blood Cell Mol. Dis. 49, 1–10. doi: 10.1016/j.bcmd.2012.03.001, PMID: 22465511 PMC4443426

[ref43] Marrink S. J. Corradi V. Souza P. C. T. Ingólfsson H. I. Tieleman D. P. Sansom M. S. P. (2019). Computational modeling of realistic cell membranes. Chem. Rev. 119, 6184–6226. doi: 10.1021/acs.chemrev.8b00460, PMID: 30623647 PMC6509646

[ref44] Moriyama R. Ideguchi H. Lombardo C. R. van Dort H. M. Low P. S. (1992). Structural and functional characterization of band 3 from southeast Asian ovalocytes. J. Biol. Chem. 267, 25792–25797. doi: 10.1016/S0021-9258(18)35679-5, PMID: 1464593

[ref45] Okawa Y. Li J. Basu A. Casey J. R. Reithmeier R. A. F. (2014). Differential roles of tryptophan residues in the functional expression of human anion exchanger 1 (AE1, band 3, SLC4A1). Mol. Membr. Biol. 31, 211–227. doi: 10.3109/09687688.2014.955829, PMID: 25257781

[ref46] Pang A. J. Reithmeier R. A. (2009). Interaction of anion exchanger 1 and glycophorin A in human erythroleukaemic K562 cells. Biochem. J. 421, 345–356. doi: 10.1042/BJ20090345, PMID: 19438409

[ref47] Popov M. Tam L. Y. Li J. Reithmeier R. A. F. (1997). Mapping the ends of transmembrane segments in a polytopic membrane protein. J. Biol. Chem. 272, 18325–18332. doi: 10.1074/jbc.272.29.18325, PMID: 9218473

[ref48] Pyle E. Kalli A. C. Amillis S. Hall Z. Lau A. M. Hanyaloglu A. C.. (2018). Structural lipids enable the formation of functional oligomers of the eukaryotic purine symporter UapA. Cell Chem. Biol. 25, 840–848.e4. doi: 10.1016/j.chembiol.2018.03.011, PMID: 29681524 PMC6058078

[ref49] Reithmeier R. A. (1979). Fragmentation of the band 3 polypeptide from human erythrocyte membranes. Size and detergent binding of the membrane-associated domain. J. Biol. Chem. 254, 3054–3060. doi: 10.1016/S0021-9258(17)30181-3, PMID: 429334

[ref50] Reithmeier R. A. (1995). Characterization and modeling of membrane proteins using sequence analysis. Curr. Opin. Struct. Biol. 5, 491–500. doi: 10.1016/0959-440X(95)80034-4, PMID: 8528765

[ref51] Reithmeier R. A. F. Casey J. R. Kalli A. C. Sansom M. S. P. Alguel Y. Iwata S. (2016). Band 3, the human red cell chloride/bicarbonate anion exchanger (AE1, SLC4A1), in a structural context. Biochim. Biophys. Acta Biomembr. 1858, 1507–1532. doi: 10.1016/j.bbamem.2016.03.030, PMID: 27058983

[ref52] Reithmeier R. A. F. Rao A. (1979). Reactive sulfhydryl groups of the band 3 polypeptide from human erythrocyte membranes. Identification of the sulfhydryl groups involved in Cu2+−o-phenanthroline cross linking. J. Biol. Chem. 254, 6151–6155. doi: 10.1016/S0021-9258(18)50531-7, PMID: 447702

[ref53] Restrepo D. Kozody D. J. Spinelli L. J. Knauf P. A. (1989). Cl-Cl exchange in promyelocytic HL-60 cells follows simultaneous rather than ping-pong kinetics. Am. J. Physiol. Cell Physiol. 257, C520–C527. doi: 10.1152/ajpcell.1989.257.3.c520, PMID: 2782393

[ref54] Sarabia V. E. Casey J. R. Reithmeier R. A. F. (1993). Molecular characterization of the band 3 protein from southeast Asian ovalocytes. J. Biol. Chem. 268, 10676–10680. doi: 10.1016/S0021-9258(18)82250-5, PMID: 8486716

[ref55] Sekler I. Kopito R. Casey J. R. (1995). High level expression, partial purification, and functional reconstitution of the human AE1 anion exchanger in Saccharomyces cerevisiae. J. Biol. Chem. 270, 21028–21034. doi: 10.1074/jbc.270.36.21028, PMID: 7673129

[ref56] Shnitsar V. Li J. Li X. Calmettes C. Basu A. Casey J. R.. (2013). A substrate access tunnel in the cytoplasmic domain is not an essential feature of the solute carrier 4 (SLC4) family of bicarbonate transporters. J. Biol. Chem. 288, 33848–33860. doi: 10.1074/jbc.M113.511865, PMID: 24121512 PMC3837127

[ref57] Simons K. Ikonen E. (1997). Functional rafts in cell membranes. Nature 387, 569–572. doi: 10.1038/42408, PMID: 9177342

[ref58] Steck T. L. (1972). Cross-linking the major proteins of the isolated erythrocyte membrane. J. Mol. Biol. 66, 295–305. doi: 10.1016/0022-2836(72)90481-0, PMID: 5038456

[ref59] Steck T. L. Fairbanks G. Wallach D. F. (1971). Disposition of the major proteins in the isolated erythrocyte membrane. Proteolytic dissection. Biochemistry 10, 2617–2624. doi: 10.1021/bi00789a031, PMID: 4104271

[ref60] Steck T. L. Ramos B. Strapazon E. (1976). Proteolytic dissection of band 3, the predominant transmembrane polypeptide of the human erythrocyte membrane. Biochemistry 15, 1153–1161. doi: 10.1021/bi00650a030, PMID: 1252433

[ref61] Stefanovic M. Markham N. O. Parry E. M. Garrett-Beal L. J. Cline A. P. Gallagher P. G.. (2007). An 11-amino acid beta-hairpin loop in the cytoplasmic domain of band 3 is responsible for ankyrin binding in mouse erythrocytes. Proc. Natl. Acad. Sci. U. S. A. 104, 13972–13977. doi: 10.1073/pnas.0706266104, PMID: 17715300 PMC1950715

[ref62] Sterling D. Reithmeier R. A. F. Casey J. R. (2001). A transport metabolon: functional interaction of carbonic anhydrase II and chloride/bicarbonate exchangers. J. Biol. Chem. 276, 47886–47894. doi: 10.1074/jbc.M105959200, PMID: 11606574

[ref63] Thurtle-Schmidt B. H. Stroud R. M. (2016). Structure of Bor1 supports an elevator transport mechanism for SLC4 anion exchangers. Proc. Natl. Acad. Sci. U. S. A. 113, 10542–10546. doi: 10.1073/pnas.1612603113, PMID: 27601653 PMC5035872

[ref64] Vince J. W. Sarabia V. E. Reithmeier R. A. F. (1997). Self-association of band 3, the human erythrocyte anion exchanger, in detergent solution. Biochim. Biophys. Acta Biomembr. 1326, 295–306. doi: 10.1016/S0005-2736(97)00033-3, PMID: 9218560

[ref65] Walter J. D. Sawicka M. Dutzler R. (2019). Cryo-EM structures and functional characterization of murine Slc26a9 reveal mechanism of uncoupled chloride transport. elife 8:e46986. doi: 10.7554/eLife.46986, PMID: 31339488 PMC6656431

[ref66] Wang C. C. Badylak J. A. Lux S. E. Moriyama R. Dixon J. E. Low P. S. (1992). Expression, purification, and characterization of the functional dimeric cytoplasmic domain of human erythrocyte band 3 in *Escherichia coli*. Protein Sci. 1, 1206–1214. doi: 10.1002/pro.5560010913, PMID: 1304397 PMC2142179

[ref67] Wang W. Tsirulnikov K. Zhekova H. R. Kayık G. Khan H. M. Azimov R.. (2021). Cryo-EM structure of the sodium-driven chloride/bicarbonate exchanger NDCBE. Nat. Commun. 12:5690. doi: 10.1038/s41467-021-25998-2, PMID: 34584093 PMC8478935

[ref68] Yu X. Yang G. Yan C. Baylon J. L. Jiang J. Fan H.. (2017). Dimeric structure of the uracil:proton symporter UraA provides mechanistic insights into the SLC4/23/26 transporters. Cell Res. 27, 1020–1033. doi: 10.1038/cr.2017.83, PMID: 28621327 PMC5539350

[ref69] Zhang Y. Chernova M. N. Stuart-Tilley A. K. Jiang L. Alper S. L. (1996). The cytoplasmic and transmembrane domains of AE2 both contribute to regulation of anion exchange by pH. J. Biol. Chem. 271, 5741–5749. doi: 10.1074/jbc.271.10.5741, PMID: 8621440

[ref70] Zhang D. Kiyatkin A. Bolin J. T. Low P. S. (2000). Crystallographic structure and functional interpretation of the cytoplasmic domain of erythrocyte membrane band 3. Blood 96, 2925–2933. doi: 10.1182/blood.V96.9.2925, PMID: 11049968

[ref71] Zhekova H. R. Pushkin A. Kayık G. Kao L. Azimov R. Abuladze N.. (2021). Identification of multiple substrate binding sites in SLC4 transporters in the outward-facing conformation: insights into the transport mechanism. J. Biol. Chem. 296:100724. doi: 10.1016/j.jbc.2021.100724, PMID: 33932403 PMC8191340

[ref72] Zhou J. Low P. S. (2001). Characterization of the reversible conformational equilibrium in the cytoplasmic domain of human erythrocyte membrane band 3. J. Biol. Chem. 276, 38147–38151. doi: 10.1074/jbc.M104333200, PMID: 11477080

